# Prevalence and associated factors of malaria in children under the age of five years in Wogera district, northwest Ethiopia: A cross-sectional study

**DOI:** 10.1371/journal.pone.0257944

**Published:** 2021-10-11

**Authors:** Adino Tesfahun Tsegaye, Andualem Ayele, Simon Birhanu

**Affiliations:** 1 Department of Epidemiology and Biostatics, Institute of Public Health, College of Medicine and Health Sciences, University of Gondar, Gondar, Ethiopia; 2 Wogera District Health Office, North Gondar Zone, Gondar, Ethiopia; 3 School of Nursing and Midwifery, Haramaya University, Harar, Ethiopia; Menzies School of Health Research: Charles Darwin University, AUSTRALIA

## Abstract

**Background:**

Malaria is a major public health problem in sub-Saharan Africa, and children are especially vulnerable. In 2019, an estimated 409,000 people died of malaria, most (274,000) were young children and 94% of the cases and deaths were in Africa. Prior studies in Ethiopia focused on the adult population and high transmission areas. Hence, this study aimed to determine the prevalence and associated factors of malaria in children under five years in low transmission areas.

**Method:**

A facility-based cross-sectional study was conducted among 585 under-five children who attended public health facilities in the Wogera district from September to October, 2017. Health facilities were selected by stratified cluster sampling, and systematic random sampling was held to select study participants from the selected facilities. Multivariable logistic regression was used to identify correlates of malaria.

**Result:**

Of 585 children who provided blood samples, 51 (8.7%) had malaria. The predominant *Plasmodium* species were *P*. *falciparum* 33 (65%) and *P*. *vivax* 18 (35%). Regularly sleeping under long-lasting insecticide treated nets (LLIN) was associated with decreased odds of malaria (AOR = 0.08, 95% CI: 0.01–0.09), and an increased odds of malaria was observed among children who live in households with stagnant water in the compound (AOR = 6.7, 95% CI: 3.6–12.6) and children who stay outdoors during the night (AOR = 5.5, 95% CI: 2.7–11.1).

**Conclusion:**

The prevalence of malaria in the study population was high. Environmental and behavioral factors related to LLIN use remain potential determinants of malaria. Continued public health interventions targeting proper utilization of bed nets, drainage of stagnant water, and improved public awareness about reducing the risk of insect bites have the potential to minimize the prevalence of malaria and improve the health of children.

## Background

In sub-Saharan Africa, infectious diseases remain the primary public health threat [1]. Malaria is one of the commonest infections, disproportionately affecting children and pregnant women. In 2019, an estimated 409,000 people died of malaria. Most (274,000) were young children, and 94% of the infections and deaths occurred in Africa [2,3]. Although several *Plasmodium* species are responsible for malaria, only a few of them cause most infections.

In 2018, *Plasmodium falciparum* accounted for 99.7% of estimated malaria cases in the World Health Organization (WHO) African Region, 50% in the WHO South-East Asia Region, 71% in the Eastern Mediterranean, and 65% in the Western Pacific. *P*. *vivax* is the predominant parasite in the WHO Region of the Americas, representing 75% of malaria cases [3]. In Ethiopia, peak malaria transmission occurs between September and December in most parts, following the rainy season from June to August, mainly affecting young children, and *P*. *falciparum* and *P*. *vivax* are the major malaria parasites [4,5].

Children under five years are one of the most vulnerable groups affected by malaria. Severe anemia, hypoglycemia and cerebral malaria are features of severe malaria more commonly seen in children than in adults [6]. Children’s susceptibility to diarrhea, respiratory infections, and other illnesses increases when they develop repeated malaria infections [7]. An estimated 2% of children who recover from cerebral malaria develop learning impairments and disabilities, including epilepsy and spasticity, resulting from the brain damage caused by the infection [8]. In general, malaria could cause severe outcomes in children in three major ways: First, since children do not usually have acquired immunity, they are more likely to develop severe malaria manifested by seizures or coma (cerebral malaria), which can cause emergency death. Second, through complications related to repeated infections such as anemia. Finally, it causes low birth weight when it happens during pregnancy and increases the risk of death in the first month of life [4].

According to the WHO 2016 report, the global prevalence of malaria among under-five children was 16% [9]. In the same year, the prevalence in Ethiopia was 0.6% [5].

The Ethiopian government developed a National Malaria Control Strategy (NMSP) for the years 2017–2020 that was envisioned to be aligned with the country’s four-year health sector transformation plan (HSTP) 2015/16–2019/20. The proposed goals for the 2017–2020 NMSP include: maintaining near-zero malaria deaths (< = 1 death per 100,000) by 2020, reducing malaria cases by 40% by 2020, and eliminating malaria from Ethiopia by 2030 [2,5].

Even though malaria is one of the leading causes of under-five morbidity and mortality in Ethiopia, prior studies focused only on the adult population and were done in malaria-endemic transmission areas. Nevertheless, it is a potential threat in non-endemic regions [5]. There has been limited information on the epidemiology of malaria among under-five children living in low malaria transmission areas [10]. This study aimed to close a critical knowledge gap by assessing the prevalence and determinants of malaria among under-five years old children living in low malaria transmission areas. The findings from this study will inform public health and clinical decision-making and will initiate further investigations.

## Methods and materials

### Study setting and design

A health facility-based cross-sectional study was conducted from September to October, 2017 in the Wogera district. Wogera is one of the districts in the North Gondar zone. It has an average altitude of greater than 2050 meters above sea level, with an estimated total population of 274,384, of which 37,152 (13.5%) are children under five years old. The district has 42 rural and one city *kebeles (the smallest administrative unit*), of which 15 *kebeles* (35.7%) are malaria-endemic. In the Wogera district, there was 1 hospital, 10 health centers, 42 health posts, and 4 private health institutions. It shares borders with Dabat and Tach-Armacho in the North, Misrak-Belesa and Janamora in the West, Merab Belesa in the South and Lay-Armacho in the East [11]. According to the new stratification of malaria risk in the country, the district is under the classification of low transmission areas with expected sporadic epidemics every five years [5]. Despite that, the report of the district health office indicates that malaria is one of the leading causes of morbidity both in adults and under-five children.

### Study participants

All children whose age was five years or below visiting the selected health facilities during the study period were the source population.

### Sample size estimation

The calculated sample size was 266 using a single population proportion formula as well as a power approach using a double proportion formula based on previous studies [12]. Adding a 10% non-response rate and a design effect of two, the final sample size was 585.

### Sampling procedure

First, we stratified the health facilities as malaria-endemic and non-endemic based on their altitude. Then, we randomly selected five health centers (Ambagiorgis HC, Gedebgie HC, Selarie HC, Tirgosgia HC, and Chichiki HC) and one hospital (Wogera hospital) from the non-endemic clusters by using a lottery method. The calculated 585 sample size was proportionally allocated to the selected health facilities. Finally, a systematic random sampling technique was used to reach under-five clients who attended the selected health facilities.

### Data collection tools and procedures

A structured questionnaire was used for data collection. The tool contained socio-demographic, environmental, and malaria prevention related questions. The questionnaire was initially developed in English and translated into Amharic for data collection. A face-to-face interview of the parents/guardians of the under-five children was conducted to collect the data.

After the interview was completed, following the Federal Democratic Republic of Ethiopia Ministry of Health National Malaria Guidelines, blood was taken from a finger prick to prepare thick and thin blood film smears [13]. Using a sterile lancet, a finger prick was performed, and 5 micro liters of whole blood was drawn from each child included in the sampling regardless of signs and symptoms of malaria using a capillary tube. The blood smears were prepared on microscope slides and stained using 10% Giemsa to be examined under 100x microscopes for the presence of malaria parasites. The thick smear was used to determine whether the malaria parasites were present or absent and the thin smear was used to identify the type of *Plasmodium* species. A positive result was defined as the presence of one or more asexual stages (trophozoite, ring stage, merozoite, or gametocyte) of *plasmodium* [14].

### Data quality assurance

Six laboratory technicians (1 from each health facility) and two supervisors from the district health office were trained for two days by the investigators. Each filled questionnaire was checked thoroughly for completeness and consistency, and the necessary feedback was given to data collectors. Recruitment was preceded by obtaining informed written consent from parents or caregivers of the children. To assure the quality of the microscopic examinations, all positive and randomly selected five percent of the negative slides were checked blindly by another experienced medical laboratory technologist.

### Operational definitions

**Bed net utilization:** was self-reported ownership and regular use of bed nets. A 15-day recall period was used to measure whether each child regularly slept under long lasting insecticide treated nets (LLIN) or not.

**Malaria**: was defined as a positive thin or thick blood film for the *Plasmodium* parasite.

### Data processing and analysis

After data collection, data were entered using Epi info version 7 and then exported to SPSS version 20 for analysis. The correlates of malaria were identified using bivariate and multivariate logistic regression models. Variables which had a P-value of <0.2 in the bivariable regression were included in the multivariable logistic regression analysis. A P-value <0.05 was considered to determine statistical significance. Finally, adjusted odds ratios (AOR) with a 95% confidence interval (CI) were used to determine the strength of association of variables.

### Ethical approval and consent to participate

Ethical approval was obtained from the ethical review committee of the Institute of Public Health, College of Medicine and Health Science, University of Gondar, Ethiopia. Permission was gained from the Amhara Regional Health Bureau, North Gondar health department, and Wogera health office. The caregivers were given detailed explanations about the study’s objectives, procedures, and potential risks and benefits, and written consent was obtained following that. The interview of each study participant took place in a separate room after the children gave blood samples. Appropriate treatment was given to children who tested positive.

## Results

### Socio-demographic characteristics of study participants

In this study, 585 children from five health centers and one district hospital participated: Gedebgie health center (HC) 178 (30.4%), Ambagiorgis HC 114 (19.5%), Tirgosgia HC 111 (19%), Selarie HC 98 (16.8%), Ambagiorgis hospital 37 (6.3%) and Chichiki HC 47 (8%). Three hundred twenty-three (55.2%) were males and 218 (37.3%) were below 12 months. About 370 (63%) of the respondents live in rural areas, and 305 (54%) of the caregivers can not read and write (Table 1).

**Table 1:** Socio-demographic characteristics of study participants, Wogera district, northwest Ethiopia, 2017.

Variable	Categories	Number (N)	Percentage (%)
Name of the health facility	Ambagiorgis Health Center	114	19.5
	Chichiki Health Center	47	8
	Gedebgie Health Center	178	30.4
	Selarie Health Center	98	16.8
	Tirgosgia Health Center	111	19
	Ambagiorgis hospital	37	6.3
Sex of the child	Female	262	44.8
	Male	323	55.2
Age of the child	<12 months	218	37.8
	12–23 months	215	36.8
	24–35 months	66	11.3
	36–47 months	56	9.6
	48–59 months	30	5.1
Residence	Rural	370	63
	Urban	215	37
Highest level of education of the parents/guardians	Illiterate	305	52
	Write and read only	134	23
	Primary	71	12
	Secondary	58	10
	Tertiary	17	3
Roof material of the household	Corrugated iron	529	90
	Grass	56	10
Wall material of the household	Mud with wood	569	97
	Wood only	16	3
Possession of a radio or TV	Yes	236	40
	No	349	60

### Indoor Residual Spraying (IRS), Long Lasting Insecticide Treated Nets (LLIN), and environmental characteristics of study participants

Only 131 (22.4%) of the respondents had LLIN. Of the respondents who possessed LLIN, 90% of respondents reported that their children had regularly slept under LLIN in the last 15 days (Table 2).

**Table 2:** IRS, LLIN, and environmental characteristics of study participants, Wogera district, northwest Ethiopia, 2017.

Variables	Categories	Number (N)	Percentage (%)
Child having regular sleeping area	Yes	260	44.4
	No	325	55.6
Child staying outside during night	Yes	33	5.6
	No	552	94.4
Incidence of IRS	Yes	48	8.2
	No	537	91.8
Possession of ITN	Yes	131	22.4
	No	454	77.6
Child sleeping under ITN regularly for the last 2 weeks	Yes	118	20.2
	No	467	79.8
Stagnant water near to the house	Yes	33	5.6
	No	552	94.4

### Magnitude of Malaria

In this study, the prevalence of malaria by microscopy among under-five children was 8.7% (51). There was a considerable variation in the prevalence rate between the health facilities, ranging from 0% at Wogera hospital to 21% at Selarie health center (Table 3).

**Table 3:** Prevalence and distribution of *Plasmodium* species, Wogera district, northwest Ethiopia, 2017.

Variables	categories	Health facilities													
		Ambagiorgis HC		Gedebgie HC		Selarie HC		Tirgosgia HC		Chichiki HC		Wogera hospital		Total	
		N	%	N	%	N	%	N	%	N	%	N	%	N	%
Malaria status	Positive	11	9.7	3	2	21	21	13	12	3	6	0	0	51	8.7
	Negative	103	90.3	175	98	77	79	98	88	44	94	37	100	534	91.3
*Plasmodium* spp. (n = 51)	*P*. *falciparum*	7	64	2	67	14	67	8	62	3	100	0	0	33	65
	*P*. *vivax*	4	36	1	33	7	33	5	38	0	0	0	0	18	35

### Factors associated with malaria infection

Both bivariable and multivariable binary logistic regression analyses were done to identify the determinants of malaria infection. In bivariate analysis, factors with a P-value of <0.2 were: place of residence, stagnant water around the home, staying outside during the night, possession of an LLIN and regularly sleeping under an LLIN for the last 2 weeks. However, place of residence, sex of the child, age of the child, age of the mother/guardian, educational status of the mother/guardian, presence of radio/television, child having a regular sleeping area, construction material of the house and incidence of IRS within six months had a P-value of >0.2 in the bivariate analysis and were not included in the final model.

In the final adjusted model, children who stayed outside at night had 5.5 times higher odds of malaria infection than children who did not stay outside at night (AOR = 5.5, 95% CI: 2.7–11.1). Children who regularly slept under a LLIN had 92% lower odds of infection than those who did not sleep regularly (AOR = 0.08, 95% CI: 0.08, 0.09). Children who lived in households with close to stagnant water had—4 times higher odds of malaria infection than children who did not live in those homes with nearby stagnant water (AOR = 4, 95% CI: 1.9, 8.1) (Table 4).

**Table 4:** Factors associated with malaria infection in under-five children, Wogera district, northwest Ethiopia, 2017.

Variables	Categories	Malaria status				Crude Odds Ratio (COR) (95% CI)	Adjusted Odds Ratio (AOR) (95% CI)
		Yes		No			
		N	%	N	%		
Place of residence	Rural	38	10	332	90	1.8 (0.9, 3.4)	1.0 (0.4, 2.2)
	Urban	13	6	202	94	1(reference)	1(reference)
Staying outside at night	Yes	43	7.8	509	92.2	8.9 (4.7, 16.8)	**5.5 (2.7, 11.1)** *
	No	8	24	25	76	1(reference)	**1(reference)**
Stagnant water	No	27	5	525	95	1(reference)	**1(reference)**
	Yes	24	72	9	28	6.7 (3.6, 12.6)	**4.0 (1.9, 8.1)** *
Possession of LLIN	No	35	7.7	419	92.3	0.6 (0.3, 1.1)	0.9 (0.2, 5.3)
	Yes	16	2.9	534	77.1	1(reference)	1(reference)
The child slept under LLIN regularly	No	38	8	429	92	1(reference)	**1(reference)**
	Yes	13	11	105	89	0.2 (0.02, 0.83)	**0.08 (0.01, 0.9)** *

*N*.*B*

* Significant at p<0.*05*.

## Discussion

In this study, we estimated the prevalence of malaria among under-five children in the low-risk area and its determinant factors, and the results showed that the malaria prevalence in under-five children was 8.7%, which is in line with the study conducted in Dilla, Southern Ethiopia, where the prevalence of malaria in under-five children was identified to be 7.1% [15] and a study of analysis of the five-year trend of malaria at Bichena primary hospital, Amhara Region, Ethiopia, where the overall prevalence of malaria was 9.28% [16].

This finding is much higher when compared to the national malaria indicator survey in 2015 that identified a prevalence of 0.6% among under-five children [5] and another study conducted in four regional states in Ethiopia, where the prevalence was 4.6% [17]. This could be due to the difference in methodology used, and also, it might be due to the season when the studies were conducted. Malaria increases from September to December (major transmission season). However, this finding is lower when compared to the global magnitude of malaria among under-five children, which is about 16% [9] and studies conducted in East Shewa 18.9% [18], Tanzania 26.3% [19], Sudan 22% [20], Uganda 19.5% [21], and Mozambique 33% [22]. Those studies were conducted in low land areas, and the difference could be due to a study population difference in the case of a study conducted in Mozambique in which the study population was people with comorbidity.

In Ethiopia, there is spatial and temporal variability in the occurrence of malaria. The current findings also demonstrated similar spatial variations in the proportion of *Plasmodium* species, with the predominant occurrence of *P*. *falciparum* infections at 65% over *P*. *vivax* at 35%. This estimate is approximately similar to the study conducted by the Carter Center in Amhara, Oromia, and Southern Ethiopia, where *P*. *falciparum* accounted for 56.5% and *P*. *vivax* for 43.5% [17], and a 7-year trend of malaria study done at primary health facilities in Northwest Ethiopia *P*. *falciparum* accounted for 15.6% of the participants, which was threefold higher than *P*. *vivax* in the seven-year trend [23]. However, other studies reported a different proportion, such as those conducted in East Shewa (*P*. *falciparum* = 41.2%, *P*. *vivax* = 57.1 and Mixed = 1.8%) [19]; Hadiya (*P*. *falciparum* = 25.5%, *P*. *vivax* = 71.7% and Mixed = 2.8%) [24] and Dilla town (*P*. *falciparum* = 26.8%, *P*. *vivax* = 62.5%, and Mixed = 10.7%) [15]. The variability could be related to the wide climatic diversity between the areas.

Sleeping under LLIN for the last two weeks was found to be protective against malaria. This evidence is supported by other similar studies conducted in East Shewa [18], Amhara, Oromia, and SNNRP [17], Dilla [15], Ethiopia [25], Ghana [26], and Uganda [21]. It was evident that using ITN properly decreased mosquito bites, and thereby decreased malaria infection.

In this study, malaria was highly prevalent among children living in households with stagnant water in the compound compared to their counterparts. This is consistent with a facility-based cross-sectional study conducted in a low transmission area of the Hadiya zone, south Ethiopia [24]. This is because water collection is one of the favorable conditions for mosquito breeding, which in turn increases malaria transmission. Staying outside during the night showed a statistically significant association with malaria. Staying outside during the night increases the probability of mosquito bites due to the nocturnal nature of the mosquito.

### Limitations of the study

As a limitation of this study, since it is a cross-sectional study, it only captures the point prevalence and can not account for seasonal trends in transmission. All surveys are self-report with no confirmation of bed net ownership or use. RDTs with PCR confirmed were not conducted, nor are there details on the life stages of detected parasites observed–gametocytemia, parasitemia.

## Conclusion

The prevalence of malaria in under-five children attending health care facilities in Wogera district was high. Regularly sleeping under a bed net, staying outside during the night, and stagnant water around the household were the main correlates of malaria. Focusing on LLIN distribution, environmental management, and changing attitudes towards malaria prevention and control through health education would help minimize the burden of malaria.

## Abbreviations

AIDS: Acquired Immune Deficiency Syndrome
AOR: Adjusted Odds Ratio
API: Annual Parasite Incidence
CI: Confidence interval
DRC: Democratic Republic of Congo
EMIS: Ethiopia Malaria Indicator Survey
HIV: Human Immune Virus
IRS: Indoor Residual Spraying
LLITN: Long Lasting Insecticide Treated Nets
OPD: Outpatient Department
RDT: Rapid Diagnostic Test
SNNPR: Southern Nation Nationalities and People Region
